# Apigenin Prevents Acetaminophen-Induced Liver Injury by Activating the SIRT1 Pathway

**DOI:** 10.3389/fphar.2020.00514

**Published:** 2020-04-24

**Authors:** Licong Zhao, Jiaqi Zhang, Cheng Hu, Tao Wang, Juan Lu, Chenqu Wu, Long Chen, Mingming Jin, Guang Ji, Qin Cao, Yuanye Jiang

**Affiliations:** ^1^ Department of Gastroenterology, Putuo Hospital, Shanghai University of Traditional Chinese Medicine, Shanghai, China; ^2^ Department of Second Clinical College, China Medical University, Shenyang, China; ^3^ Experiment Center for Science and Technology, Shanghai University of Traditional Chinese Medicine, Shanghai, China; ^4^ Shanghai University of Medicine & Health Sciences of Integrated Traditional Chinese and Western Medicine, Shanghai University of Traditional Chinese Medicine, Shanghai, China; ^5^ Institute of Digestive Diseases, Longhua Hospital, Shanghai University of Traditional Chinese Medicine, Shanghai, China

**Keywords:** apigenin, acetaminophen-induced liver injury, SIRT1 pathway, flavonol, antioxidant, SIRT1-p53 axis, inflammatory response, oxidative stress

## Abstract

Acetaminophen (APAP) overdose is the main cause of acute liver failure. Apigenin (API) is a natural dietary flavonol with high antioxidant capacity. Herein, we investigated protection by API against APAP-induced liver injury in mice, and explored the potential mechanism. Cell viability assays and mice were used to evaluate the effects of API against APAP-induced liver injury. Western blotting, immunofluorescence staining, RT-PCR, and Transmission Electron Microscope were carried out to determine the signalling pathways affected by API. Analysis of mouse serum levels of alanine/aspartate aminotransferase (ALT/AST), malondialdehyde (MDA), liver myeloperoxidase (MPO) activity, glutathione (GSH), and reactive oxygen species (ROS) revealed that API (80 mg/kg) owned protective effect on APAP-induced liver injury. Meanwhile, API ameliorated the decreased cell viability in L-02 cells incubated by APAP with a dose dependent. Furthermore, API promoted SIRT1 expression and deacetylated p53. Western blotting showed that API promoted APAP-induced autophagy, activated the NRF2 pathway, and inhibited the transcriptional activation of nuclear p65 in the presence of APAP. Furthermore, SIRT1 inhibitor EX-527 reduced protection by API against APAP-induced hepatotoxicity. Molecular docking results indicate potential interaction between API and SIRT1. API prevents APAP-induced liver injury by regulating the SIRT1-p53 axis, thereby promoting APAP-induced autophagy and ameliorating APAP-induced inflammatory responses and oxidative stress injury.

## Introduction

Acetaminophen (APAP, paracetamol) is a widely used antipyretic and analgesic non-prescription drug. It is generally safe and effective, but long-term and/or large doses will lead to severe acute liver injury that can eventually develop into liver failure and even death (Nourjah et al., 2006; Fontana, 2008). Approximately 2,600 people are reportedly hospitalized each year due to overdose of APAP, 56,000 people are seen in the emergency room, and ~500 people die (Lee, 2008). In China, due to the population, medical conditions, and medical knowledge, a large number of high-dose APAP cases occur annually, and liver damage has become a serious health problem (Shen et al., 2019). In the human body, the main cause of liver damage is due to APAP consuming glutathione (GSH) in the liver, causing cellular oxidative stress and liver damage (Kanno et al., 2017). A high dose of APAP produces N-acetyl-P-phenylpropanimide (NAPQI) under oxidation of the cytochrome P450 enzyme system. NAPQI is a highly toxic X-ray product that can be used in combination with glutathione to deplete APAP. Binding to proteins in hepatocytes forms protein conjugates, causing intracellular oxidative stress, mitochondrial and DNA damage, eventually leading to liver necrosis (Walker et al., 2017).

4',5,7-trihydroxyflavone (Apigenin, API, Versulin) is a well-known flavonoid isolated from *Matricaria chamomilla.* In our previous research, we found that flavonoids can protect against various liver diseases, especially acetaminophen-induced liver damage, *via* anti-oxidation and anti-inflammatory mechanisms (Jing et al., 2018; Shi et al., 2018; Zhao et al., 2019). API can protect against various liver injuries caused by alcohol (Wang et al., 2017), lipopolysaccharide (Zhou et al., 2017), ischemia/reperfusion (Tsaroucha et al., 2016), and CCl_4_ (Simeonova et al., 2014). Furthermore, API can protect against liver injury in an APAP mouse model, but its specific mechanism of action remains unknown (Yang et al., 2013).

Sirtuin 1 (SIRT1) regulates protein deacetylation, participates in protein transcription and translation, regulates cell proliferation, oxidative stress, and metabolism, and plays an important role in metabolic diseases, tumors, and cardiac function (Consiglio et al., 2014; Cui et al., 2016; Qin et al., 2016). Rada et al. found that overexpression of SIRT1 ameliorated hepatoxicity induced by APAP, and inhibits inflammation responses and oxidative stress (Rada et al., 2018). A previous study also reported that SIRT1 suppresses p53 acetylation in ischemia/reperfusion liver injury (Nakamura et al., 2017). However, the mechanism in APAP liver injury is still unknown. Furthermore, the structure of resveratrol (RSV) indicates that RSV molecules may modulate the interaction between the 7-amino-4-methylcoumarin peptide and the extended N-terminal domain of SIRT1, and promote SIRT1 activity (Cao et al., 2015; Naini et al., 2019). However, the correlation between apigenin and SIRT1 is still not clear.

Herein, we investigated apigenin protection mechanisms against APAP-induced liver injury. We also investigated the participation of SIRT1 in this process.

## Materials and Methods

### Drugs and Reagents

Apigenin (purity >99.5%) was purchased from Shanghai Hitsanns Co., Ltd (Shanghai, China). Kits for alanine aminotransferase (ALT), aspartate aminotransferase (AST), malondialdehyde (MDA), myeloperoxidase (MPO), and glutathione (GSH) were purchased from Nanjing Jiancheng Bioengineering Institute (Nanjing, China). H2DCFDA, RPMI1640, and fetal bovine serum (FBS) were purchased from Life Technology (Carlsbad, CA, USA). A Pierce BCA Protein Assay Kit was purchased from Thermo Fisher Scientific (Waltham, MA, USA). EX-527 was purchased from Sigma-Aldrich (St. Louis, MO, USA). Whole-cell protein extraction kits and enhanced chemiluminescence kits were obtained from Millipore (Darmstadt, Germany). Antibodies for immunoblotting, including β-actin (#4970), Lamin B (#13435), SIRT1 (#8469), p53 (#2524), ac382-p53 (#2525), NRF2 (#12721), and p65 (#8242) were purchased from Cell Signaling Technology (Danvers, MA, USA; all 1: 1,000 dilutions). Enzyme-linked immunosorbent assay (ELISA) kits were purchased from RapidBio (West Hills, CA, USA). TRIzol reagent was purchased from Life Technology (Carlsbad, CA, USA). PrimeScript RT Master Mix and SYBR Premix Ex Taq were purchased from TaKaRa (Shiga, Japan). APAP, NAPQI, 3-(4,5-dimethyl-thiazol-2-yl)2,5-diphenyltetrazolium bromide (MTT), and other reagents were purchased from Sigma-Aldrich unless otherwise indicated.

### Experimental Animals

C57BL/6 mice (20 ± 2 g) were purchased from Shanghai Laboratory Animal Center (Shanghai, China) and fed according to guidelines approved by the Experimental Animal Ethical Committee of Shanghai University of Traditional Chinese Medicine. They were raised in a constant temperature and humidity room (22 ± 1°C, 65 ± 5% humidity) with standard diet and water. The protocol was reviewed and approved by the Experimental Animal Ethical Committee of Shanghai University of Traditional Chinese Medicine (Permit Number: PZSHUTCM190315014).

### Animal Treatment

Forty mice were randomly divided into five groups; (1) vehicle control, (2) APAP (400 mg/kg), (3) APAP (400 mg/kg) + API (20 mg/kg), (4) APAP (400 mg/kg) + API (80 mg/kg), and (5) API (80 mg/kg). Mice were pre-administered orally with API (20 or 80 mg/kg per day) for 7 consecutive days. On the last day, mice were orally administered a single dose of APAP (400 mg/kg) after administration of API for 1 h. Animals were sacrificed 6 h after APAP intoxication and plasma and liver tissues were collected.

To assess the role of SIRT1 in regulating APAP-induced liver injury, 48 mice were randomly divided into six groups; (1) Dimethyl sulfoxide (DMSO) (2) DMSO + APAP (400 mg/kg), (3) DMSO + APAP (400 mg/kg) + API (80 mg/kg), (4) EX-527 (10 mg/kg), (5) EX-527 (10 mg/kg) + APAP (400 mg/kg), and (5) EX-527 (10 mg/kg) + APAP (400 mg/kg) + API (80 mg/kg). Mice were pre-administered orally with API (80 mg/kg per day) or injected intraperitoneally with DMSO or EX-527 (10 mg/kg) for 7 consecutive days. On the last day, mice were orally given a single dose of APAP (400 mg/kg) after administration of API for 1 h. Animals were sacrificed 6 h after APAP intoxication and plasma and liver tissues were collected.

### Biochemical Analysis for Blood and Liver

Blood and livers were withdrawn after cardiac puncture. Blood samples were kept at room temperature for 2 h and then centrifuged at 840 g for 10 min to collect serum. Part of liver tissue was fixed in formalin and the rest was snap-frozen in liquid nitrogen and stored at -80°C. ALT, AST in serum and MDA, MPO, ROS, GSH in liver were measured by kits, according to manufacturer's protocols (Nanjing Jiancheng Bioengineering Institute, Nanjing, China).

### Liver Histological Observation

Liver histological was observed to evaluate liver damage degree and structural changes under a light microscope (Olympus, Tokyo, Japan) after fix, dehydration, paraffin embedded, section, and H&E staining.

### Cell Culture

The L-02 cell line was derived from an adult human normal liver (Cohen et al., 1997) (Cell Bank, Type Culture Collection of Chinese Academy of Sciences, Shanghai). L-02 cells were cultured in RPMI1640 supplemented with 10% [v/v] fetal bovine serum, 2 mM glutamine, 100 U/ml penicillin, and 100 mg/ml streptomycin, and incubated at 37°C in a humidified atmosphere containing 5% CO_2_.

### Cell Viability Assay

L-02 cells were plated into 96-well plates at an initial density of 5,000 cells per well. Cells were pre-incubated with EX-527 or DMSO for 15 min, then incubated with or without API for another 15 min after attachment, and finally incubated with APAP or NAPQI for different time periods. After treatment, cells were incubated with 500 μg/ml MTT for 4 h. Blue formazan was dissolved in 10% SDS-5% isobutanol-0.01M HCl, and plates were scanned on a microplate reader (Thermo Scientific) at 570 nm with 630 nm as a reference. Cell viability was normalised as a percentage of control wells.

### RNA Isolation and Quantitative Real-Time PCR (qRT-PCR)

RNA was isolated using TRIzol reagent (Invitrogen) according to manufacturer's instructions and reverse-transcribed using a miScript Reverse Transcription Kit. qRT-PCR was performed using a SYBR Premium Ex Taq II kit (TaKaRa) on an ABI PRISM 7500 Sequence Detection System (Applied Biosystems). All reactions were performed in triplicate and the mean value was used to calculate expression levels after normalisation against β-actin.

### Protein Extraction and Western Blot Analysis

L-02 cells were lysed using RIPA buffer and protein concentration was determined using a BCA protein assay kit. Approximately 30 μg of protein from each sample was separated by 10% sodium dodecyl sulphate-polyacrylamide gel electrophoresis (SDS-PAGE) and transferred to a polyvinylidene fluoride (PVDF) membrane. Membranes were blocked with 5% skimmed milk in TBST and incubated with primary antibodies overnight at 4°C. Membranes were incubated with the corresponding secondary antibody for 1 h at room temperature and washed in TBST. Protein signals were detected using Super ECL Plus Detection Reagent.

### Enzyme-Linked Immunosorbent Assay (ELISA)

The levels of inflammatory cytokines IL-6, TNF-α, and MCP-1 in blood were determined with an ELISA kit (Nanjing Jiancheng Bioengineering Institute), according to standard protocols.

### Immunofluorescence Analysis

Immunofluorescence staining was performed as previously reported (Shah et al., 2015). Briefly, cells were washed twice with 0.01 M PBS for 15 min followed by addition of proteinase K and blocking solution. Primary antibodies, as described in the above paragraph (1:100 in PBS), were added and incubated at 4°C overnight. After washing with PBS, secondary antibodies (FITC- and TRITC-conjugated, Santa Cruz Biotechnology, 1:50 in PBS) were then applied at room temperature for an additional 90 min. Slides were washed twice with PBS for 5 min, 4',6-diamidino-2-phenylindole (DAPI) was applied to stain nuclei, and glass cover slips were mounted on slides with mounting medium. Images were captured using a FluoView FV 1000 confocal microscope (Olympus, Japan).

### Molecular Docking

A molecular docking study was performed to investigate interactions between API and SIRT1 using Autodock vina 1.1.2 (Trott and Olson, 2010). The three-dimensional (3D) coordinates of SIRT1 (PDB ID: 5BTR) were retrieved from the RCSB Protein Data Bank. The 3D structure of API was drawn using ChemBioDraw Ultra 14.0 and converted to a 3D structure by ChemBio 3D Ultra 14.0. The AutoDockTools 1.5.6 package (Sanner, 1999; Morris et al., 2009) was employed to generate docking input files. The binding site of SIRT1 was identified as centre_x: -20.454, centre_y: 58.86, and centre_z: 9.056 with dimensions size_x: 15, size_y: 15, and size_z: 15. To increase the accuracy of the calculation, the value of exhaustiveness was set to 20, and default parameters were used unless otherwise mentioned. The best-scoring pose as judged by Vina docking score was chosen and further analyzed using PyMoL 1.7.6 software.

### Statistical Analysis

Data were expressed as the mean ± standard error of the mean (SEM). Significant differences were determined by One-Way ANOVAs with LSD *post hoc* tests; and P < 0.05 is considered to be statistically significant.

## Results

### Protective Effect of API *In Vivo*

**Figure 1:**
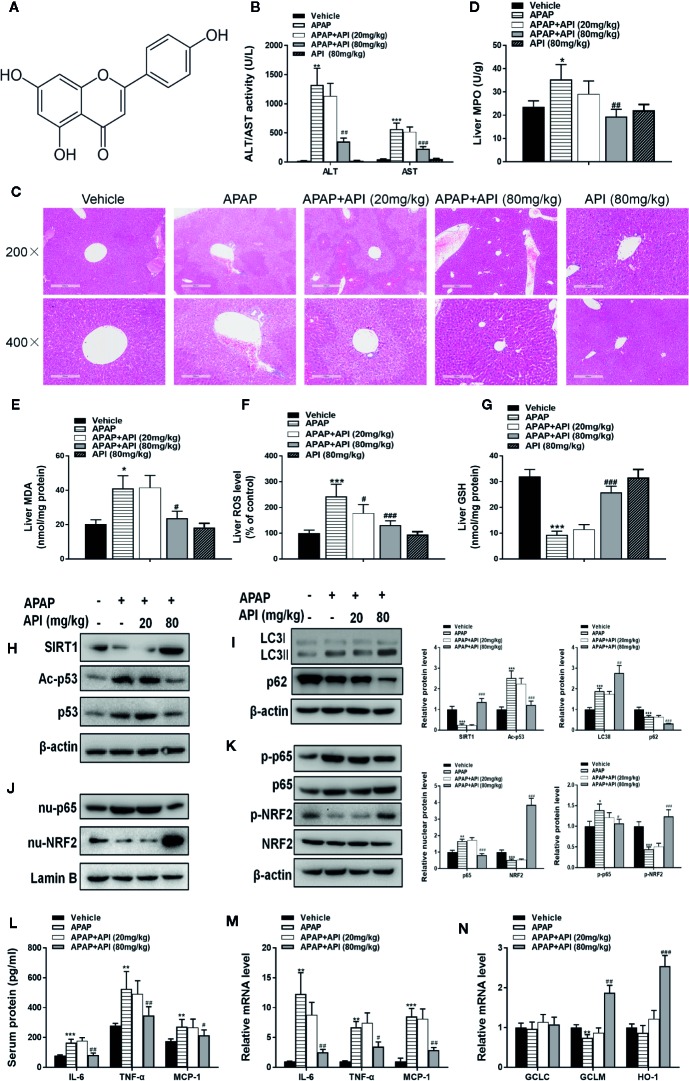
Protective effect of apigenin (API) *in vivo*. **(A)** Structural formula of API. The molecular formula and weight of API is C_15_H_10_O_5_ and 270.24 g/mol. **(B)** Serum alanine aminotransferase (ALT) and aspartate aminotransferase (AST) activities at 6 h after administration with acetaminophen (APAP). **(C)** Typical images of H&E staining of liver segments (×200 and ×400 magnification). **(D)** Myeloperoxidase (MPO) activity in mice liver tissue was detected. **(E)** Reactive oxygen species (ROS) levels in mice liver tissue was detected. **(F)** Malondialdehyde (MDA) activity in mice liver tissue was detected. **(G)** Glutathione (GSH) levels in mice liver tissue was detected. **(H)** Liver protein levels of SIRT1 and Acetyl-p53 determined by western blot. **(I)** Liver protein levels of LC3-II/I assessed by western blot. **(J)** Protein expression of p65 and nuclear localisation of NRF2 determined by western blot and quantified. **(K)** Phosphorylation of NRF2 and p65 determined by western blot and quantified. **(L)** Serum protein concentrations of IL-6, TNF-α, and MCP-1 determined by enzyme-linked immunosorbent assay (ELISA) in mice. **(M)** Liver mRNA levels of IL-6, TNF-α, and MCP-1 determined by real-time PCR (RT-PCR). **(N)** Liver mRNA levels of GCLC, GCLM, and HO-1 determined by RT-PCR. Data are expressed as mean ± SEM (n = 8; ^*^ *p* < 0.05, ^**^ *p < *0.01, ^***^ *p < *0.001 compared to vehicle; ^#^ *p* < 0.05, ^##^ *p < *0.01, ^###^ *p < *0.001 compared to APAP).

APAP significantly increased the levels of ALT/AST in mouse serum, whereas they were decreased significantly in the presence of API (**Figure 1B**) at 6 h and mice treated with API alone did not display any obvious differences compared to the vehicle group. It was found in H&E staining results that intrahepatic haemorrhage and destruction of liver structures occurred in APAP group, whereas these symptoms were ameliorated by API. This observation further confirmed the protective effects of API against APAP-induced liver injury in mice (**Figure 1C**). Next, we found APAP lead the increasement of MPO, MDA, and ROS. while administration of API suppressed the level of MPO, MDA, and ROS (**Figures 1D–F**). In addition, API improved GSH levels in mouse liver compared with APAP (**Figure 1G**). These results indicated that API alleviated APAP-induced Next, APAP decreased protein expression levels of SIRT1 and promoted p53 acetylation, while administration of API promoted protein expression of SIRT1 and inhibited p53 acetylation (**Figure 1H**). **Figure 1I** showed that APAP up-regulated the expression of LC3-II/I (autophagy-related proteins) in liver tissues, whereas treatment with API appeared to promote expression of these proteins in liver tissues. Furthermore, inflammatory response and oxidative stress-related proteins were detected by western blot. **Figures 1J, K** showed that API promoted the phosphorylation of NRF2 and increased the level of nuclear NRF2, which indicated that API induced the transcriptional activation of NRF2. Heme oxygenase 1 (HO-1) and catalytic or modified subunits of GCL are downstream genes regulated by NRF2 (Kaspar et al., 2009). As shown in **Figure 1N**, APAP suppressed mRNA levels of GCLM, and APAP showed no significant effect on mRNA levels of GCLC or HO-1. Moreover, API increased the mRNA levels of GCLM and HO-1 significantly. Furthermore, API inhibited phosphorylation of p65 and decreased the level of nuclear p65 increased by APAP (**Figures 1J, K**). ELISA results showed that concentrations of IL-6, TNF-α, and MCP-1 in serum increased significantly in the presence of APAP (**Figure 1L**). Additionally, **Figure 1M** showed that API reversed the increased mRNA levels induced by APAP.

### API Prevents APAP-Induced Cytotoxicity *In Vitro*

**Figure 2:**
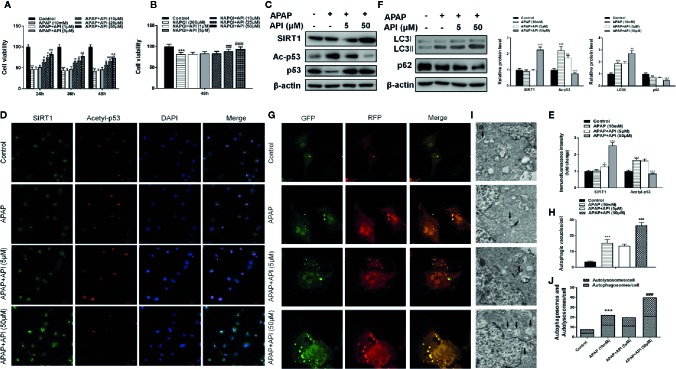
Protective effect of apigenin (API) *in vitro*. **(A, B)** Cell viability evaluated by 3-(4,5-dimethyl-thiazol-2-yl)2,5-diphenyltetrazolium bromide (MTT) assay in response to acetaminophen (APAP) (10 mM) or N-acetyl-P-phenylpropanimide (NAPQI) (200 μM) exposure in different time periods in L-02 cells. **(C)** The level of SIRT1 and acetyl-p53 proteins determined by western blotting with APAP (10 mM) for 48 h in L-02 cells and quantified. **(D, E)** Representative photomicrographs of SIRT1 (green) and acetyl-p53 (red) immunofluorescence. DAPI was used to counterstain nuclei. **(F)** Expression of LC3-II/I proteins determined by western blot with APAP (10 mM) for 48 h in L-02 cells and quantified. **(G, H)** Typical images of immunofluorescence staining of mRFP-GFP-LC3 in L-02 cells cultured APAP (10 mM) for 48 h. Typical profiles of autophagosomes (RFP + GFP + dots) and autolysosomes (RFP + GFP - dots) per cell section assessed by confocal microscopy are shown and quantified. **(I, J)** Autophagic vacuoles (autophagosomes) determined by transmission electron microscopy (TEM) with APAP (10 mM) for 48 h. Representative TEM images are shown, and typical autophagosomes are marked with black arrows. The number of autophagosomes per cell was calculated by counting the number of double-membrane organelles in 10 cells. The experiments were performed three times. Data are expressed as mean ± SEM (*p < 0.05, **p < 0.01, ***p < 0.001 compared to controls; ^#^ *p* < 0.05, ^##^ *p < *0.01, ^###^ *p < *0.001 compared to APAP).

As shown in **Figures 2A, B**, APAP (10 mM) and NAPQI (200 µM; a metabolic product of APAP) both decreased cell viability in L-02 cells. API reversed the suppression of cell viability caused by APAP and NAPQI in L-02 cells in a concentration-dependent manner. Next, western blot results indicated no obvious change in SIRT1 protein levels compared with control and APAP groups in L-02 cells. After treatment with API, protein levels of SIRT1 were significantly increased. API also reversed APAP-induced p53 acetylation in L-02 cells (**Figure 2C**). Immunofluorescence analysis of SIRT1 and acetyl-p53 ace in L-02 cells confirmed these results (**Figures 2D, E**).

**Figure 3:**
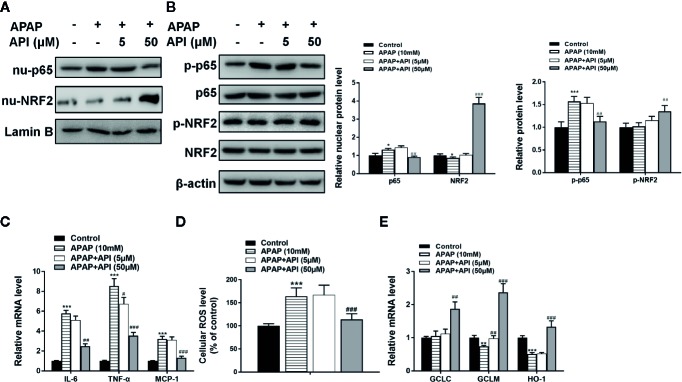
Apigenin (API) ameliorates acetaminophen (APAP)–induced inflammatory responses and oxidative stress injury in L-02 cells. **(A)** Nuclear p65 and nuclear NRF2 protein levels determined by western blot with APAP (10 mM) for 48 h in L-02 cells and quantified. **(B)** Phosphorylation of NRF2 and p65 proteins determined by western blot with APAP (10 mM) for 48 h in L-02 cells and quantified. **(C)** mRNA levels of IL-6, TNF-α, and MCP-1 determined by quantitative real-time PCR (qRT-PCR) with APAP (10 mM) for 48 h in L-02 cells. **(D)** Cellular ROS levels in L-02 cells. **(E)** mRNA levels of GCLC, GCLM, and HO-1 determined by qRT-PCR with APAP (10 mM) for 48 h in L-02 cells. The experiments were performed three times. Data are expressed as mean ± SEM (*p < 0.05, **p < 0.01, ***p < 0.001 compared to control; ^#^ *p* < 0.05, ^##^ *p* < 0.01, ^###^ *p <* 0.001 compared to APAP.

Furthermore, western blotting showed that API enhanced the APAP-induced increases in LC3-II/I, suggesting that API promoted APAP-induced autophagy in L-02 cells (**Figure 2F**). Immunofluorescence analysis revealed increased levels of autolysosomes (red) and autophagosomes (green) in L-02 cells incubated with APAP for 48 h, and more significantly with API (**Figures 2G, H**). Transmission electron microscopy (TEM) of autophagic vacuoles in L-02 cells confirmed these results (**Figures 2I, J**). Furthermore, inflammatory response and oxidative stress-related proteins were detected by western blotting in L-02 cells. **Figures 3A, B** indicated that API promoted the phosphorylation of NRF2 and increased the levels of nuclear NRF2, suggesting that API induced the transcriptional activation of NRF2.

**Figure 3D** showed that APAP increased cellular ROS levels in L-02. Meanwhile, API (50 μM) inhibited enhanced ROS levels induced by APAP in L-02 cells. Furthermore, APAP suppressed mRNA levels of GCLM and HO-1. APAP had no significant effect on mRNA levels of GCLC. Moreover, API increased the mRNA levels of GCLC, GCLM, and HO-1 significantly, compared with the APAP group (**Figure 3E**). **Figures 3A**, B showed that API inhibited the phosphorylation of p65 and decreased the level of nuclear p65 increased by APAP. Meanwhile, APAP increased mRNA levels of IL-6, TNF-α, and MCP-1 in L-02 cells, while API reversed these APAP-induced increases (**Figure 3C**). Accordingly, these results suggested that API up-regulated SITR1, promoted autophagy, and ameliorated inflammatory responses and oxidative stress *in vitro*.

### API Prevents Hepatotoxicity *Via* SIRT1 *In Vitro*

**Figure 4:**
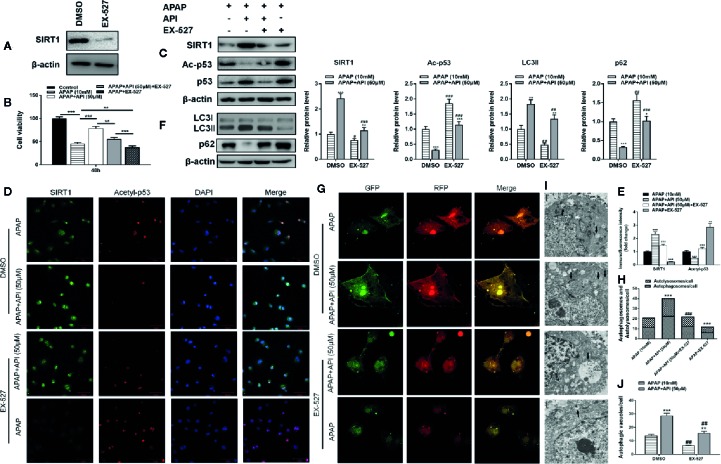
SIRT1 inhibitor EX-527 reverses the protective effects of apigenin (API) against acetaminophen (APAP)–induced cytotoxicity in L-02 cells. **(A)** Protein levels of SIRT1 determined by western blot with EX-527 (10 μM) for 48 h in L-02 cells. **(B)** Cell viability assessed by 3-(4,5-dimethyl-thiazol-2-yl)2,5-diphenyltetrazolium bromide (MTT) assay in response to APAP (10 mM) exposure for 48 h in L-02 cells. **(C)** Protein levels of SIRT1 and acetyl-p53 determined by western blot with APAP (10 mM) for 48 h in L-02 cells and quantified. **(D, E)** Representative photomicrographs of SIRT1 (green) and acetyl-p53 (red) immunofluorescence. DAPI was used to counterstain nuclei. **(F)** Protein levels of LC3-II/I determined by western blot with APAP (10 mM) for 48 h in L-02 cells and quantified. **(G, H)** Representative images of immunofluorescence staining of mRFP-GFP-LC3 in L-02 cells with APAP (10 mM) for 48 h. Representative profiles of autophagosomes (RFP + GFP + dots) and autolysosomes (RFP + GFP - dots) per cell section assessed by confocal microscopy are shown and quantified. **(I, J)** Autophagic vacuoles (autophagosomes) detected by transmission electron microscopy (TEM) with APAP (10 mM) for 48 h. Representative TEM images are shown and typical autophagosomes are marked with black arrows. The number of autophagosomes per cell was calculated by counting the number of double-membrane organelles in 10 cells. The experiments were performed three times. Data are expressed as mean ± SEM [*p < 0.05, **p < 0.01, ***p < 0.001 compared to APAP; ^#^ *p < *0.05, ^##^ *p < *0.01, ^###^ *p < *0.001 compared to dimethyl sulfoxide 9(DMSO)].

To explore whether API plays a role *via* the SIRT1 pathway, we used SIRT1 inhibitor EX-527 to inhibit SIRT1 expression. As expected, western blot data showed that EX-527 (10 μM) significantly down-regulated SIRT1 expression in L-02 cells (**Figure 4A**). MTT assessment showed that the hepatocyte-protective effect of API was inhibited by treatment with EX-527, and a single treatment with EX-527 aggravated cell injuries induced by APAP (**Figure 4B**). Western blot results also indicated that EX-527 reversed inhibition of p53 acetylation caused by API in L-02 cells (**Figure 4C**). Immunofluorescence analysis of SIRT1 and acetyl-p53 in L-02 cells confirmed these results (**Figures 4D, E**).

**Figure 5:**
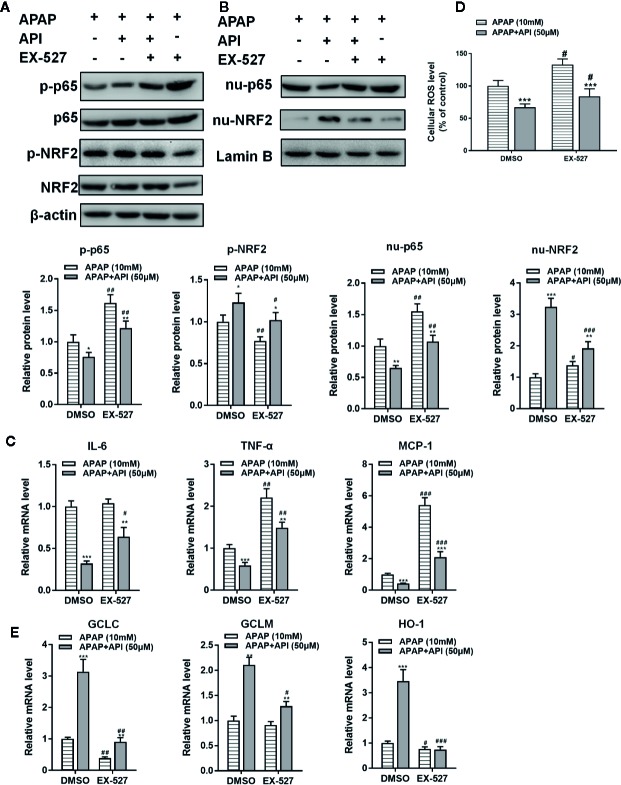
SIRT1 inhibitor EX-527 reverses the anti-inflammation and antioxidant effects of apigenin (API) in L-02 cells. **(A)** Nuclear localisation of p65 and NRF2 determined by western blot with acetaminophen (APAP) (10 mM) for 48 h in L-02 cells and quantified. **(B)** Protein levels and phosphorylation of NRF2 and p65 determined by western blot with APAP (10 mM) for 48 h in L-02 cells and quantified. **(C)** mRNA levels of IL-6, TNF-α, and MCP-1 assessed by real-time PCR (RT-PCR) with APAP (10 mM) for 48 h in L-02 cells. **(D)** Reactive oxygen species (ROS) levels in L-02 cells. **(E)** mRNA levels of GCLC, GCLM, and HO-1 determined by RT-PCR with APAP (10 mM) for 48 h in L-02 cells. The experiments were performed three times. Data are expressed as mean ± SEM [*p < 0.05, **p < 0.01, ***p < 0.001 compared to APAP; ^#^ *p < *0.05, ^##^ *p < *0.01, ^###^ *p < *0.001 compared to dimethyl sulfoxide (DMSO)].

To clarify the relationship between SIRT1 and autophagy, we analyzed protein levels of LC3-II/I by western blot. The results suggested that EX-527 inhibited autophagy induced by API and APAP in L-02 cells (**Figure 4F**). Immunofluorescence staining and TEM also showed that EX-527 reversed the autophagy effects induced by API and APAP (**Figures 4G−J**). Analysis of phosphorylation and nuclear localisation of NRF2 showed that EX-527 repressed transcriptional activation of NRF2 induced by API (**Figures 5A, B**). Furthermore, **Figure 5D** showed that EX-527 up-regulated ROS levels in L-02 cells, which were decreased by API. In addition, qRT-PCR indicated that EX-527 suppressed mRNA levels of GCLM, GCLC, and HO-1 significantly (**Figure 5E**).

We then measured the levels of p65 in L-02 cells. Western blots revealed that EX-527 promoted the phosphorylation of p65 and nuclear translocation of p65 in L-02 cells (**Figures 5A, B**). Meanwhile, EX-527 significantly increased mRNA levels of IL-6, TNF-α, and MCP-1 in L-02 cells (**Figure 5C**). From these observations, it would appear that the SIRT1 inhibitor EX-527 reversed the protective effects of API by promoting p53 acetylation, inhibiting autophagy, and aggravating inflammatory responses and oxidative stress in L-02 cells.

### Theoretical Binding Mode of API and SIRT1

**Figure 6:**
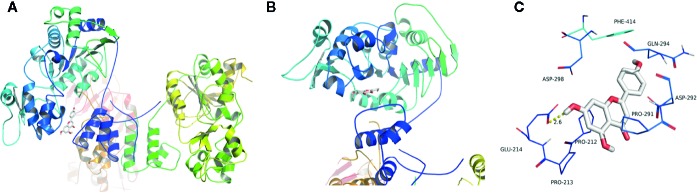
Theoretical binding mode of apigenin (API) in the binding site of SIRT1. **(A)** Front view of the docking mode of API (white) in the binding site of SIRT1 (shown in ribbon representation and coloured by structural elements). **(B)** Top view of the docking mode of API (white) in the binding site of SIRT1 (presented in ribbon representation and colored by structural elements). **(C)** Representative amino acid residues surrounding API (white) in the binding pocket of SIRT1.

**Figures 6A**, B represented the theoretical binding mode of apigenin in the binding site of SIRT1. API is positioned in the hydrophobic pocket, surrounded by residues Phe-414, Pro-291, Pro-212, and Pro-213, forming a stable hydrophobic binding region (**Figures 6A, B**). Detailed analysis indicates that the B ring of API forms a CH–π interaction with the side chain of residue Phe-414. Furthermore, anion-π interactions were observed between the A ring of API and residues Asp-298 and Glu-214 (**Figure 6C**). Notably, the carbonyl oxygen of the hydroxyl group at C-7 of A ring of API forms hydrogen bonding interactions with residue Glu-214 (bond length: 2.6 Å). These interactions helped API to anchor in the binding site of SIRT1. In addition, the estimated binding energy of API is -7.4 kcal/mol, suggesting that API is an agonist of SIRT1. In summary, the above molecular simulations elucidated the interactions between API and SIRT1, providing valuable information for the further development of SIRT1 agonists.

### API Prevents APAP-Induced Liver Injury by Up-Regulating SIRT1 *In Vivo*

**Figure 7:**
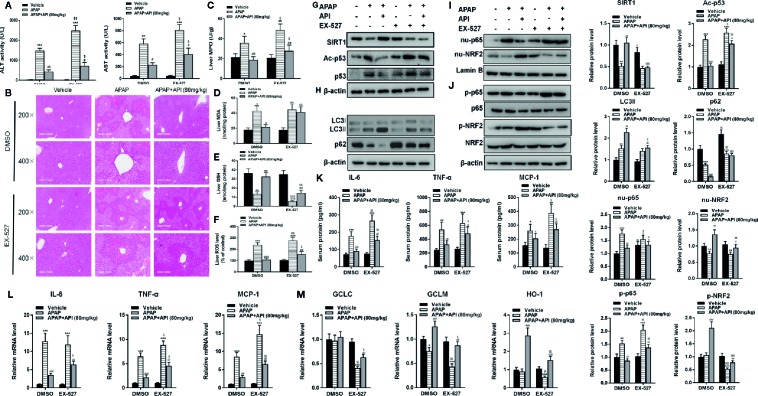
Apigenin (API) prevents acetaminophen (APAP)–induced liver injury by promoting SIRT1 *in vivo.* **(A)** Serum alanine/aspartate aminotransferase (ALT/AST) activities was detected. **(B)** Representative images of H&E staining of mouse liver sections (×200 and ×400 magnification). **(C)** Myeloperoxidase (MPO) activity in mice liver tissue was detected. **(D)** Malondialdehyde (MDA) activity in mice liver tissue was detected. **(E)** Glutathione (GSH) levels in mice liver tissue was detected. **(F)** Reactive oxygen species (ROS) levels in mice liver tissue was detected. **(G)** Liver protein levels of SIRT1 and acetyl-p53 determined by western blot and quantified. **(H)** Liver protein levels of LC3-II/I determined by western blot and quantified. **(I)** Nuclear localisation of p65 and NRF2 by western blot and quantified. **(J)** Liver protein levels and phosphorylation of NRF2 and p65 determined by western blot and quantified. **(K)** Serum protein concentrations of IL-6, TNF-α, and MCP-1 determined by enzyme-linked immunosorbent assay (ELISA) in mice. **(L)** Liver mRNA levels of IL-6, TNF-α, and MCP-1 assessed by quantitative real-time PCR (qRT-PCR). **(M)** Liver mRNA levels of GCLC, GCLM, and HO-1 determined by qRT-PCR. Data are expressed as mean ± SEM [n = 8; ^*^ *p <*0.05, ^**^ *p <*0.01, ^***^ *p <*0.001 compared to vehicle; ^#^ *p <*0.05, ^##^ *p <*0.01, ^###^ *p <*0.001 compared to APAP, ^$^ *p <*0.05, ^$$^ *p <*0.01, ^$$$^ *p <*0.001 compared to dimethyl sulfoxide (DMSO)].

To check if the results for EX-527 could be recapitulated in mice, H&E staining, ALT/AST activities, and liver MPO, ROS, MDA, and GSH levels were examined. All results were consistent with the above *in vitro* data; API protected against APAP-induced liver damage by promoting SIRT1 (**Figures 7A−F**). Western blot results indicated that EX-527 (10 mg/kg) inhibited levels of SIRT1 and significantly promoted p53 acetylation in mouse liver compared with DMSO (**Figure 7G**). We also analyzed protein levels of LC3-II/I by western blot, and found that EX-527 down-regulated LC3-II/I, which indicated that EX-527 inhibited autophagy in mouse liver (**Figure 7H**). Additionally, analysis of phosphorylation and nuclear localisation of NRF2 showed that EX-527 suppressed transcriptional activation of NRF2 induced by API (**Figures 7I, J**). Furthermore, RT-PCR indicated that EX-527 repressed mRNA levels of GCLM, GCLC, and HO-1 significantly (**Figure 7M**). Next, we assessed levels of p65 in mouse liver, and western blot results revealed that EX-527 promoted the phosphorylation of p65 and nuclear translocation of p65 in mouse liver (**Figures 7I, J**). Meanwhile, EX-527 increased mRNA levels and concentrations of IL-6, TNF-α, and MCP-1 in mouse liver, and concentrations of IL-6, TNF-α, and MCP-1 in mouse serum (**Figures 7K, L**). These results showed that API prevented APAP-induced liver damage by regulating the SIRT1/p53 axis and promoting autophagy, thereby inhibiting inflammatory responses and oxidative stress *in vivo*.

## Discussion

Apigenin (API), widely found in plants and vegetables, is believed to exert anti-tumor, anti-oxidation, anti-inflammatory, and other pharmacological effects (Arango et al., 2015; Venigalla et al., 2015; Feng et al., 2016; Sung et al., 2016). Yang et al. showed the protective effect of API against APAP by enhancing hepatic GSH in mice model (Yang et al., 2013). However, it is well known that within the hepatocyte, APAP is metabolized to NAPQI by CYP450 enzymes. NAPQI (a metabolite of APAP) can deplete cellular GSH in the liver, and lead to oxidative stress-induced liver injury (Jaeschke et al., 2003; Jaeschke et al., 2012). It was unclear whether API alleviated the cytotoxicity of NAPQI or only inhibited APAP metabolism in Yang's article. Hence, the effect of API on APAP drug metabolism should be excluded. In our study, we used the metabolism product of APAP, NAPQI to validate the protective effect of API. The results showed NAPQI suppressed the cell viability of L-20 cells, while with the treatment of API, this inhibition was reversed (**Figure 2B**). This indicated the API alleviated the cytotoxicity of NAPQI directly.

In our current research, we revealed that SIRT1 is the target of API which API could directly bind to. The accumulated evidence indicated that the SIRT1-p53 axis is one of the critical targets in liver failure (Wang et al., 2015; Kulkarni et al., 2016; Nakamura et al., 2017), but its role in liver development is not yet known. The current research suggested that SIRT1 directly interacts with API, and stimulation by API may represent an innovative therapeutic strategy by which SIRT1 may deacetylate p53, promote autophagy, inhibit oxidative stress, and reduce inflammatory responses induced by acetaminophen in a mouse model and in L-02 cells. Importantly, treatment with SIRT1 inhibitor EX-527 aggravated liver injury and reversed the therapeutic action of API both *in vivo* and *in vitro*. Consistent with previous studies, we found that direct treatment of hepatocytes, such as L-02 or primary mouse hepatocytes with acetaminophen did not alter the level of SIRT1 (Mobasher et al., 2013). Hepatoxicity induced by APAP was accompanied by an inflammatory response containing stimulation of resident macrophages like Kupffer cells (Liu and Kaplowitz, 2006; Laskin, 2009; Williams et al., 2011). Patricia et al. (Rada et al., 2018) found that conditioned media from RAW 264.7 macrophages co-cultured with APAP reduced protein levels of SIRT1 in hepatocytes. Thus, in future studies, we will focus on the correlation between Kupffer cells, APAP and API.

Previous studies demonstrated that NRF2 and the NFκB pathway are related to APAP-induced liver injury, and flavonoids can target these pathways to ameliorate hepatoxicity (Zheng et al., 2015; Pang et al., 2016; Shi et al., 2018). The SIRT1-p53 axis can modulate oxidative stress and inflammatory responses *via* NRF2 and the NFκB pathway (Yang et al., 2015; Yoon et al., 2016; Khan et al., 2018). Though the antioxidant capacity of API was discovered to contribute to its inhibition on APAP-induced hepatotoxicity (Yang et al., 2013), whether the anti-inflammatory activity of API would similarly contribute to its defense against APAP-induced liver injury is still unknown. In our research, we found that API induced NRF2 and suppressed NFκB activation. In addition, the oxidative stress parameters like MDA amount and ROS formation and inflammatory indices like IL-6, TNF-α, and MCP-1 proved that the alleviation of API against APAP-induced liver oxidative stress and inflammation response injury occurred both *in vitro* and *in vivo*.

Previous studies reported that SIRT1 could deacetylated p53 (Luo et al., 2001; Vaziri et al., 2001). SIRT1 specially deacetylated p53 at the K382 acetylation site and negatively regulated the capacity of p53 to promote the level of target genes (Luo et al., 2001; Cheng et al., 2003). The suppression of SIRT1 by a specific inhibitor like EX-527 could induce p53 hyperacetylation and enhance p53-dependent transcriptional activity (Lain et al., 2008). It is found in our study that API promoted SIRT1 activity and deacylated p53, which up-regulated both the NRF2 pathway and glutathione levels, accompanied by inhibition of ROS production, and this simultaneously down-regulated the NFκB pathway and the secretion of pro-inflammatory cytokines. After using EX-527, an inhibitor of SIIRT1, the acetylation of p53 was increased which caused the effect of API reversed both *in vitro* and *in vivo*.

Autophagy preforms a vital role in survival mechanisms in response to adverse intracellular events. The autophagy mechanism selectively removes damaged organelles, especially mitochondria. Hence, autophagy defends against cell injury induced by mitochondria (Lum et al., 2005; Tan et al., 2018). Excessive acetaminophen can lead to hepatoxicity by inducing mitochondrial injury and autophagy. Interestingly, promoting autophagy attenuated APAP-induced liver cell death by removing damaged mitochondria (Ni et al., 2012; Lin et al., 2014; Yan et al., 2018). After inhibiting autophagy, APAP-mediated hepatoxicity is aggravated. However, rapamycin, an autophagy promoter, could alleviate APAP-induced liver injury (Ni et al., 2012). Earlier research reported the effect of API on autophagy. Fang et al. found that API promoted autophagy and alleviated myocardial oxidative and inflammatory injury, and endotoxin-induced myocardial toxicity (Li et al., 2017). Additionally, API plays an anti-tumor role by promoting autophagy in various tumors such as skin cancer (Bridgeman et al., 2016), colon cancer (Lee et al., 2014; Chen et al., 2019), thyroid carcinoma (Zhang et al., 2015), and hepatocellular carcinoma (Gao et al., 2018; Yang et al., 2018). Moreover, SIRT1 can directly deacetylate major proteins of autophagy, like Atg5 and Atg8, remove them from the inhibitory state, and thus stimulating autophagy (Ma et al., 2016). At the same time, SIRT1 could also promote autophagy by deacetylating p53 (De et al., 2018). In the present study, we showed that API could interact with SIRT1 and thereby activate autophagy, which reversed damage caused by APAP in hepatocytes.

## Conclusions

**Figure 8:**
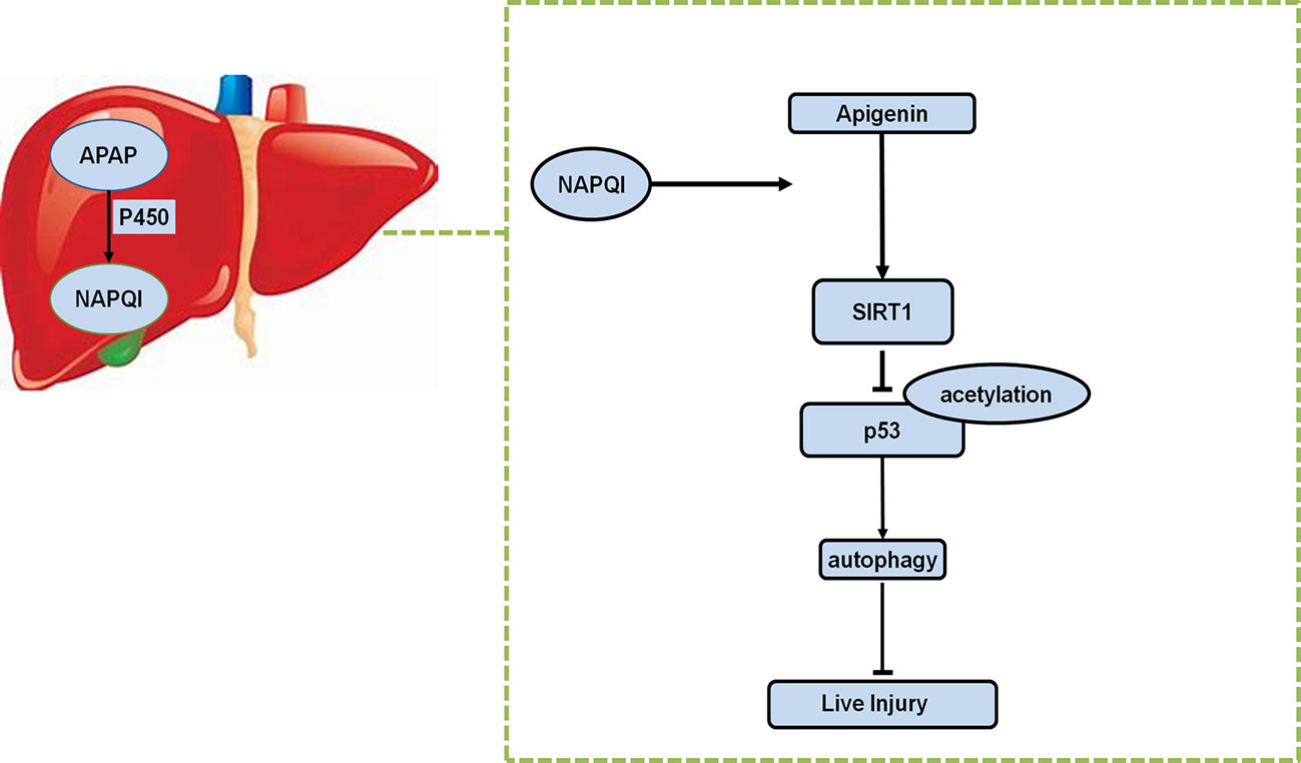
Schematic representation of proposed mechanism of API on the prevention of APAP-induced liver injury.

In conclusion, we revealed that API promotes autophagy through the SIRT1-p53 axis, and ameliorates oxidative stress and inflammation responses, potentially reversing liver-mediated APAP injury (**Figure 8**).

## Data Availability Statement

The datasets generated for this study are available on request to the corresponding authors.

## Ethics Statement

The animal study was reviewed and approved by Experimental Animal Ethical Committee of Shanghai University of Traditional Chinese Medicine.

## Author Contributions

LZ, JZ, and CH: performed the research. GJ, QC, YJ: designed the research study. TW, JL, and CW: contributed essential reagents or tools. LC and MJ: collected the data. JZ and CH: analyzed the data. LZ and JZ: wrote the paper.

## Funding

This work was financially supported by National Natural Science Foundation of China (81703879); Clinical research project of Shanghai Municipal Commission of health (201840377, 201940449); Putuo District of Shanghai Science And Technology Commission Research Project(ptkwws201813, ptkwws201806); Key specialties of Putuo Hospital Affiliated to Shanghai University of Traditional Chinese Medicine(2016103A); the Budget Project of Shanghai University of Traditional Chinese Medicine (2016YSN60); and the Budget of Experiment Center for Science and Technology (18LK022)

## Conflict of Interest

The authors declare that the research was conducted in the absence of any commercial or financial relationships that could be construed as a potential conflict of interest.
